# The Role of Nonerythroid Spectrin *α*II in Cancer

**DOI:** 10.1155/2019/7079604

**Published:** 2019-05-02

**Authors:** Anne Ackermann, Angela Brieger

**Affiliations:** Medical Clinic I, Biomedical Research Laboratory, University Clinic Frankfurt, Frankfurt am Main, Germany

## Abstract

Nonerythroid spectrin *α*II (SPTAN1) is an important cytoskeletal protein that ensures vital cellular properties including polarity and cell stabilization. In addition, it is involved in cell adhesion, cell-cell contact, and apoptosis. The detection of altered expression of SPTAN1 in tumors indicates that SPTAN1 might be involved in the development and progression of cancer. SPTAN1 has been described in cancer and therapy response and proposed as a potential marker protein for neoplasia, tumor aggressiveness, and therapeutic efficiency. On one hand, the existing data suggest that overexpression of SPTAN1 in tumor cells reflects neoplastic and tumor promoting activity. On the other hand, nuclear SPTAN1 can have tumor suppressing effects by enabling DNA repair through interaction with DNA repair proteins. Moreover, SPTAN1 cleavage products occur during apoptosis and could serve as markers for the efficacy of cancer therapy. Due to SPTAN1's multifaceted functions and its role in adhesion and migration, SPTAN1 can influence tumor growth and progression in both positive and negative directions depending on its specific regulation. This review summarizes the current knowledge on SPTAN1 in cancer and depicts several mechanisms by which SPTAN1 could impact tumor development and aggressiveness.

## 1. Background

Nonerythroid spectrin *α*II (SPTAN1, also termed *α*-Fodrin) is a cytoskeletal protein that belongs to the family of spectrins. The spectrin family includes several structural proteins (*α*- and *β*-spectrin, *α*-actinin, dystrophin, and utrophin) that build and stabilize the cytoskeleton by forming a hexagonal mesh under the plasma membrane and ensuring stability and organization of organelles in the cell [1, 2]. Antiparallel heterodimers of *α*- and *β*-spectrin form tetramers in a head to head arrangement, which allow stabilization of interacting partners and serve as a structural platform for various transmembrane proteins including channels, receptors, and transporters [3–6].

In humans, spectrin isoforms are encoded by two *α*- and five *β*-spectrin genes [4, 7]. *α*-spectrin, exclusively expressed in erythroid cells, is termed erythroid spectrin *α*I (SPTA1), while SPTAN1 represents the ubiquitous form in all other cell types. Human SPTA1 and SPTAN1 only share 58% sequence identity and differ markedly in their carboxy-terminal sequence [8, 9]. Both can be expressed as different isoforms through alternative splicing from one gene locus [9–11]. As this heterogeneity occurs at the C-terminus near potential calcium and actin-binding domains, it is possible that different isoforms fulfill different functions [9]. Alternatively spliced forms of SPTAN1 were also identified to be unique in different tissues [11].

**Figure 1:**
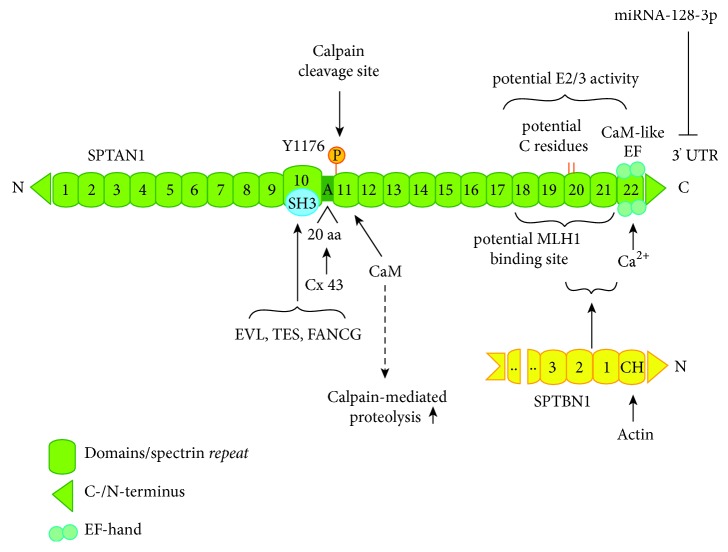
*Structure of SPTAN1*. SPTAN1 harbors 22 domains, which are presented in green. Shown are the following: characteristic spectrin repeats (green boxes); C- and N-terminus (green rectangle); domain 10 with a SH3 domain (blue circle), which allows binding of EVL, TES, and FANCG; a 20 amino acid (aa) motif and alternatively spliced region between domains 10 and 11, which allows specific binding of Connexin 43 (Cox43); phosphorylation site (orange) at residue Y1176 in domain 11 and behind that the calpain cleavage site of SPTAN1 and the calmodulin (CaM) binding site, which regulates calpain-mediated proteolysis; a potential MLH1 binding site between domains 18 and 22; potential cystein (C) residues (orange lines) in domain 20, which might mediate a potential E2/E3 ubiquitin-protein-conjugating or -ligating activity; domains 20 and 21, which mediate dimerization of SPTAN1 and SPTBN1 by binding to the N-terminal first two spectrin repeats of SPTBN1 (yellow) [5] and C-terminal the CaM-like domain 22, which can bind calcium through two EF-hand motifs. Translational inhibition by miRNA-128-3p targets the 3′ untranslated region (3′UTR) of SPTAN1 [16]. SPTBN1 can bind actin through its N-terminal Calponin homology (CH) domain [4]. Spectrin heterodimers formed by antiparallel lateral dimerization of SPTAN1 and SPTBN1 then form tetramers by head to head assembly [3, 4]. Modified after Bennett and Baines (2001) and Baines (2010) [4, 17].

SPTAN1 is mapped to chromosome 9q33 -> q34, encompasses 7,787 nucleotides, and encodes for a 2,472 amino acid protein with a predicted molecular weight of 284 kDa [9]. The protein contains 22 domains, of which domains 1-9 and 11-21 comprise the spectrin typical triple helical repeats consisting of 106 amino acids (Figure 1). Domain 10 is a src homology domain 3 (SH3) motif known to be involved in cytoskeletal interactions [4, 7]. Interactions of spectrin repeats are diverse and defy any classification of their preferred interaction site but are crucial wherever they are identified [12]. Due to their unique binding properties, spectrin repeats can have important roles in assembly of complex and multiprotein structures involved in cytoskeletal architecture as well as in forming large signal transduction complexes [12]. Domain 11 harbors a cleavage site for calpain (also termed calcium-activated protease 1, CDP-1) and a calmodulin-binding site [13, 14]. The C-terminal domain 22 is related to calmodulin and can bind calcium through two EF-hand calcium-binding motifs [1, 4, 15]. These EF-hand motifs are juxtaposed to the actin-binding domain on the adjacent *β*-spectrin subunit (Figure 1) [4]. Hence, both calpain and calmodulin might enhance actin-binding capacities of spectrin.

**Figure 2:**
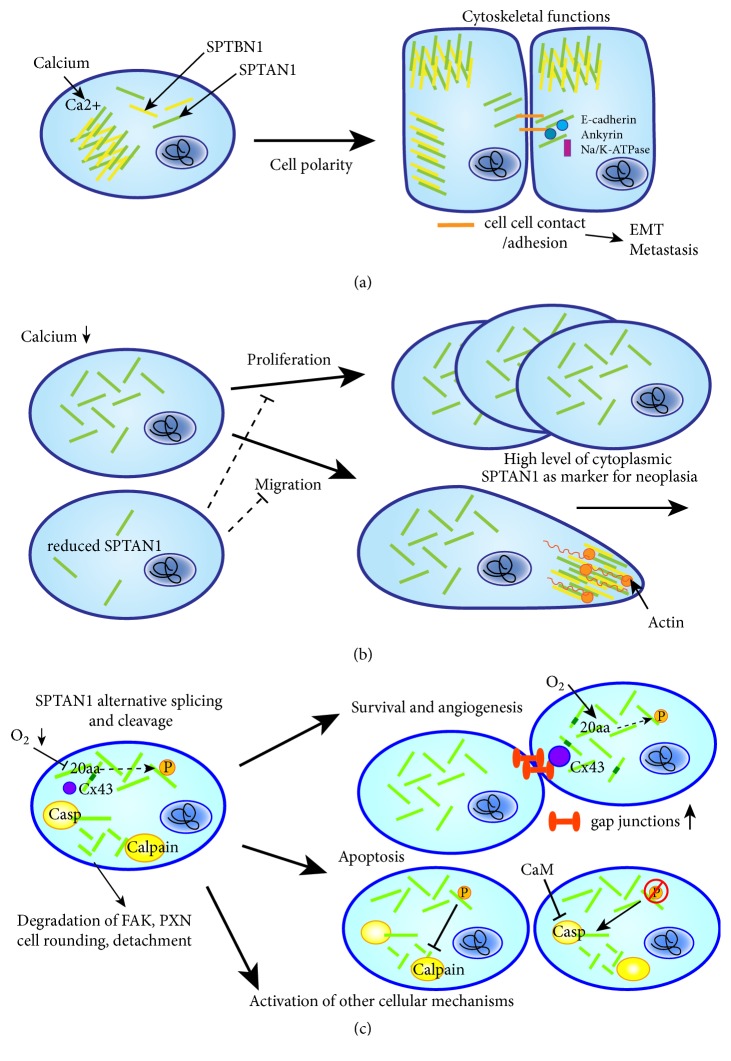
*Localization and functional relevance of cytoplasmic SPTAN1*. SPTAN1 can have diverse functions in the cell depending on its localization. (a) SPTAN1 (green bars) serves as cytoskeletal scaffolding protein and, under physiological calcium levels, together with SPTBN1 (yellow bars) and other proteins, it forms a stabilizing mesh beneath the cell membrane allowing cell polarity. Shown are epithelial cells expressing SPTAN1 apically and laterally in the cell. Upon cell-cell contact (orange bars), SPTAN1 interacts with E-cadherin, ankyrin, and Na/K-ATPase and thus might influence EMT and metastasis. (b) SPTAN1 can affect tumor growth and outcome by enhancing cell proliferation and migration (upper panel), which are impaired in case of reduced SPTAN1 levels (lower panel). High levels of cytoplasmic SPTAN1 in proliferating cells could be used as marker for neoplasia. Moreover, migration might be influenced by the interaction of SPTAN1 and SPTBN1 with actin filaments. (c) SPTAN1 has also a role in survival, angiogenesis, apoptosis, and other cellular mechanisms through expression of alternative spliced forms and cleavage products. A SPTAN1 spliceform including a 20 amino acid (aa) motif contributes to gap junctions (orange dumbbells) through association of this motif with Connexin 43 (Cx43). This association is regulated by JNK-mediated phosphorylation. Expression of this spliceform is repressed during hypoxia, thus leading to a decrease in gap junctions. SPTAN1 is involved in apoptosis if cleaved by calpain and caspases. This is regulated by phosphorylation of Y1176 and dephosphorylation enhances the proteolytic susceptibility of SPTAN1 to calpain and caspases 2, 3, and 7. Cleavage leads to membrane blebbing and irreversible cell death. Caspase-mediated cleavage can be inhibited by calmodulin (CaM) binding and indicates again the influence of calcium homeostasis. SPTAN1 cleavage products can further lead to cell rounding and detachment by degrading FAK and paxillin (PXN) and can activate further yet unknown cellular mechanisms.

**Figure 3:**
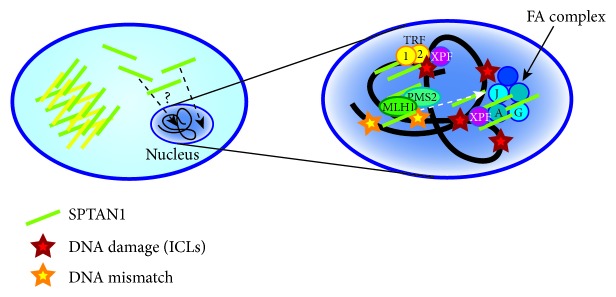
*SPTAN1 in DNA repair*. SPTAN1 can translocate into the nucleus. In the nucleus, SPTAN1 can interact with various partners including proteins involved in DNA repair and fanconi anemia (FA). By binding FA proteins in nuclear complexes, SPTAN1 is stabilized and might act as scaffold to align or enhance DNA repair associated proteins at sites of damage. The FA protein FANCG (G) is able to bind to SPTAN1 through its SH3 domain. Via FANCA (A) and XPF, SPTAN1 enables DNA repair of interstrand crosslinks (ICLs). SPTAN1 circumvents telomere dysfunction after ICL damage by interaction with TRF1/2 and XPF. Furthermore, SPTAN1 can directly interact with MLH1, which mediates DNA mismatch repair (MMR). FANCJ, which is required for correction of the cross-link response, also interacts with the MMR complex MutL*α*, consisting of MLH1 and PMS2. A SPTAN1-dependent DNA repair mechanism, however, still remains unknown.

Due to their role as scaffolding proteins, spectrins interact with numerous different binding partners and therefore fulfill multiple functions such as organization of a cytoskeleton underlying the plasma membrane and regulation of the activity of transmembrane proteins they interact with. Together with nonerythroid spectrin *β*II (SPTBN1), SPTAN1 can influence the cytoskeleton organization by interacting with membrane-associated proteins including ankyrin, protein 4.1, and adducin as well as the actin cytoskeleton [3, 18]. In addition, SPTAN1 can also modulate different ion channels and other involved proteins [4, 7, 19]. Besides its function as a cytoskeletal scaffolding protein (Figure 2(a)), the ubiquitous expression of SPTAN1 indicates additional important functions for this protein, which has been described in cell mechanisms including development, cell shape, cell-cell contact, apoptosis, cell adhesion, and cell cycle [20–23]. Furthermore, SPTAN1 is not confined to the plasma membrane but can also be distributed throughout the cell where it may execute various functions. In the nucleus (Figure 3), SPTAN1 was shown to interact with different proteins involved in DNA repair, chromatin remodeling, and fanconi anemia (FA) and with transcription factors, indicating that it potentially affects various critical cellular pathways [24, 25]. Further functions have been proposed for spectrins including control of cell proliferation, a role in protein sorting and trafficking, control of cell division, and transcription activity, which remain yet unknown for SPTAN1 but are intriguing avenues to investigate for a broader understanding of this protein biology [7].

In line with the functional diversity of SPTAN1, it is not surprising that SPTAN1 appears to play a role in tumorigenesis. The hallmarks of cancer include sustaining proliferative signaling, evading growth suppressors, activating invasion and metastasis, inducing angiogenesis, and resisting cell death and meanwhile have been extended to deregulation of cellular energetics, genome instability, and mutations [26, 27]. Therefore, proteins involved not only in one but many tumor types and in a broad spectrum of cancer characteristics, features, and mechanisms are of highest interest, as they might serve as potential biomarkers or even as predictors of therapeutic response.

Due to the wide range of SPTAN1's actions, it can potentially influence several or even every step from tumor development to progression and metastasis. Therefore, it is vital to better understand its involvement in various functions and mechanisms affecting cancer.

By combining the current knowledge of SPTAN1 in cancer and illustrating potential mechanisms for SPTAN1 influence on tumor development, progression, patient outcome, and therapeutic response, this review aims to summarize existing data about the role of SPTAN1 in carcinogenesis. However, there are still many unanswered questions and further investigations are mandatory to better understand the role of SPTAN1 as a potential neoplasia, tumor, and therapeutic response marker.

## 2. SPTAN1 in Various Cancer Types

Until now, changes in the expression of SPTAN1 have been described in a variety of tumors and tissues but defy any clear classification. SPTAN1 rather seems to have opposite effects in different tumors. On one hand, the majority of data suggest overexpression of SPTAN1 in cancer and progression. On the other hand, reduced expression of SPTAN1 has also been observed in tumors.

In light of a decrease in SPTAN1 expression, we observed in MLH1-deficient tumors and similar to data recently described for the membrane-associated skeletal protein adducin [45], we propose that SPTAN1 might function two-sided as a tumor suppressor or promotor. Studies that have investigated expression and mechanisms of action of SPTAN1 in tumors have allowed gaining some insights into its role in cancer. They are summarized in Table 1.

### 2.1. SPTAN1 in Colorectal Cancer

Overexpression of SPTAN1 in cancer was first described in 1989 in sporadic colorectal cancer (CRC) by Younes et al. and has been reported to promote tumorigenesis [28]. Interestingly, increased cytoplasmic SPTAN1 was detectable not only in colon adenomas and carcinomas but also in Crohn's disease and tumor environment and other epithelial neoplasms including adenocarcinomas of breast, stomach, and small intestine, suggesting enhanced SPTAN1 level as a nonspecific marker for neoplasia of both benign and malignant origin [28]. A model established for SPTAN1 in cancer assumed increased apical SPTAN1 as a reaction to pathological stress at the brush border, whereas increased cytoplasmic levels marked neoplastic activity [28]. Whether rearrangements in SPTAN1 localization are dependent on actin filaments and whether SPTAN1 exists on membranes or in the cytoplasm with or without SPTBN1 remain under speculation. Additionally, the predictive value of SPTAN1 expression to distinguish neoplastic tissue in samples and biopsies is still under discussion.

In 2013, a proteogenomic analysis of human CRC cell lines representing different pathological stages identified SPTAN1 and SPTBN1 as potential markers for tumor and metastases state [46]. Whereas SPTBN1 was reduced, SPTAN1 expression was increased in moderately invasive and poorly differentiated CRC compared to nonpolyposis cancer cell lines [46].

By comparing SPTAN1 expression level in DNA mismatch repair- (MMR-) deficient and MMR-proficient colorectal cancer or other cell lines, our group could demonstrate that loss of the MMR protein MLH1 was correlated with a significant reduction of SPTAN1 expression [29]. Since previously performed two hybrid experiments showed interaction of SPTAN1 with MLH1, the connection between loss of MLH1 and SPTAN1 reduction might be explained by lack of interaction and therefore destabilization of SPTAN1 in absence of MLH1 in MLH1-deficient cell lines [47].

Very recently our group analyzed the connection between SPTAN1 and MLH1 in a large cohort of CRCs and we observed clearly enhanced SPTAN1 level in sporadic CRCs compared to normal adjacent mucosa, while MLH1-deficient sporadic CRCs or Lynch syndrome tumors showed a visible reduction in SPTAN1 expression [30]. In addition, we could demonstrate that downregulation of SPTAN1 expression via shRNA resulted in reduced cell-cell contact, impaired cell proliferation, and decreased migration* in vitro* [30]. The observed association of MLH1 status with SPTAN1 expression in CRC suggests a predictive value for SPTAN1 as a marker for cancer development and progression.

In clinical practice, MMR-deficient tumors show better clinical outcome and less metastasis than tumors with functional MMR [48]. The exact reasons for this divergent behavior are still unclear, but SPTAN1 might play an important role, as reduced SPTAN1 levels have been shown to significantly impair proliferation and migration in different CRC cell lines [29]. Interestingly, when patient outcome and metastasis were correlated with SPTAN1 expression, a decline in SPTAN1 levels was found with increasing tumor stage and metastatic status, which accentuates the divergent role of SPTAN1 [30].

### 2.2. SPTAN1 in Gastric Cancer

Until now, only two studies have described SPTAN1 expression in gastric cancer [31, 32]. In the first study, published in 2002, Lee and coworkers identified SPTAN1 as a differentially expressed gene in gastric cancer using cDNA microarrays [31]. Since the data showed that SPTAN1 was enhanced in the intestinal type of gastric cancer, Lee et al. suggested SPTAN1 as a marker for classifying gastric cancers. This was confirmed two years later by Zhang et al. showing that SPTAN1 gene expression was significantly higher in gastric cancer tissue as well as dysplastic tissue than in normal mucosa [32].

### 2.3. SPTAN1 in Lung Cancer

In lung cancer, SPTAN1 was first described in 1994 by Sormunen et al. who found more intense staining and expression of SPTAN1 in all types of lung carcinomas compared to normal tissue [33]. Strong intracytoplasmic and membrane-associated staining in tumors was observed not only for SPTAN1 but also for the multifunctional, filamentous protein actin. They suggested that the diffuse distribution of SPTAN1 features undifferentiated reserve cells and reflects a high proliferative capacity.

Twenty years later, SPTAN1 again became a gene of interest in lung cancer when it was identified by exome and mRNA sequencing in lung adenocarcinoma [34]. The data showed that in never-smokers SPTAN1 harbors recurrent mutations and correlates with pathway deregulation and worse clinical outcome [34]. However, in this case, SPTAN1 was reduced in tumors compared to normal lungs and could indicate impaired DNA repair [34]. Whether this is due to the identified mutations in SPTAN1 still remains unclear. Interestingly,* in vitro* data of lung cancer cells demonstrated that SPTAN1 is suppressed by microRNA-128-3p, which led to enhanced sensitivity to cytostatic mitomycin C (MMC) by limiting DNA repair capacity [16].

### 2.4. SPTAN1 in Leukemia

In leukemia cell lines, enhanced expression of heterodimeric SPTAN1/SPTBN1 was shown to be induced by dimethyl sulfoxide (DMSO) treatment followed by local rearrangement of this protein complex [49]. In contrast, Hashida et al. only saw a slight increase in SPTAN1/SPTBN1 but major changes in actin during myeloid leukemia cell differentiation and therefore concluded that this heterodimer did not have a major function in actin-induced cell motility [50]. However, alternative pathways of SPTAN1 function besides actin-mediated cell structuring seem increasingly likely.

In 2017, the first SPTAN1 fusion gene was described in an atypical chronic myeloid leukemia (aCML) patient [42]. At the RNA level, C-terminal SPTAN1 including an incomplete spectrin repeat and the EF-hand domain was fused to colony-stimulating factor 3 receptor (CSF3R), which is frequently mutated in aCML. The affected patient showed poor response to src kinase inhibitor therapy with Dasatinib, suggesting that the fusion transcript could not be sufficiently inhibited and instead kept activating distinct signaling pathways [42]. Binding of calcium via the EF-hand domain of SPTAN1 and a resulting conformational and functional change could contribute to this activity. However, this hypothesis needs further clarification.

### 2.5. SPTAN1 in Other Cancer Types

Regarding the expression of SPTAN1 in other tumor entities, little has been published so far.

In breast cancer, altered expression and upregulation of membranous and cytoplasmic SPTAN1 were observed in two independent studies, in 1992 and 1999 [35, 36]. In particular high-grade tumors showed cytoplasmic accumulation of SPTAN1, which positively correlated with p53 expression [36].

In bladder cancer, SPTAN1 was identified in recurrence-associated gene signatures and suggested as a predictor of disease recurrence at an early tumor stage [37]. Significant changes in SPTAN1 were observed in the group of patients without recurrence [37].

Until now, no data regarding the expression level of SPTAN1 in prostate carcinomas are available. However, reduced SPTAN1 expression was found in a lung metastasis of a prostate cancer patient [38]. In this study, SPTAN1 was identified as a suitable candidate for the prediction of prostate tumor progression and suggested as a potential biomarker [38].

In cutaneous tumors of various origins, loss of membrane-associated SPTAN1 was detected, whereas cytoplasmic staining of SPTAN1 was increased and associated with less differentiated, invasive cells of these tumors [39]. Basal as well as squamous cell carcinomas and malignant melanomas display increasing invasion and metastatic capacities, probably reflected by the different patterns of SPTAN1 expression. This supports the concept that the absence or decrease of membrane-associated SPTAN1 is essential for proliferation and increased cytoplasmic SPTAN1 during invasion [39]. In melanomas, however, some cells were strongly stained, while others were completely negative for SPTAN1. Therefore, the expression of SPTAN1 in melanomas must be clarified by further investigation.

Looking at soft tissue tumors (SFTs), increasing SPTAN1 level and more aggressive tumor behavior were also described [40]. The expression of SPTAN1, identified by gene expression profiling, was higher in more aggressive types of desmoid-type fibromatosis and malignant mesenchymal tumors compared to benign mesenchymal tumors. The study suggests SPTAN1 as a marker and/or target in SFTs functionally related to locally aggressive tumors. Curiously, elevated SPTAN1 expression could not be confirmed at the RNA and protein level and no association of SPTAN1 with the metastatic potential of SFTs could be shown. This might be due to a different metastatic pattern of sarcomas compared to carcinomas, which metastasize rather to the lung and liver than to lymph nodes, and missing information on the role of SPTAN1 in this regard [40].

## 3. SPTAN1 in Cancer Development and Progression

As described in detail above, a change of SPTAN1 expression level has been found in various tumor entities. In particular, SPTAN1 seems to have an important impact on cancer development and progression by various mechanisms.

In 2000, Gascard and Mohandas [7] suggested the idea of cytoskeletal proteins including spectrin as key players in signal transduction pathways, by anchoring or regulating kinases and their corresponding proteins [7]. SPTA1 was demonstrated to interact with protein kinase C and tyrosine kinase [51, 52]. In addition, SPTAN1 has been shown to interact via its SH3 domain with phosphoproteins participating in actin assembly, including Ena/vasodilator-stimulated phosphoprotein-like protein (EVL) [53] and vasodilator-stimulated phosphoprotein (VASP) which induces apoptosis by SPTAN1 breakdown depending on the VASP phosphorylation status (Figure 1) [54, 55]. The observed interaction of SPTAN1 with EVL and the potential tumor suppressor Tes fits well in this functional context and might furthermore influence cell-cell contacts and focal adhesions [3, 56].

### 3.1. SPTAN1 as a Tumor Promoter

As already mentioned, SPTAN1 has been mostly shown to be upregulated in tumors compared to normal mucosa (Table 1) [28, 32, 33, 35, 36, 39–41]. In particular, SPTAN1 expression was increased heterogeneously in the cytoplasm, whereas membrane bound SPTAN1 partly disappeared as described in cutaneous tumors [39]. This localization change is probably due to a switch of SPTAN1's function.

Whereas membranous SPTAN1 can act as a cytoskeletal scaffold by interacting with associated proteins and thereby ensures cell polarity, its distribution throughout the cell allows SPTAN1 access to other potential interacting partners and may enhance cell growth and cell proliferation [28]. Therefore, increase of cytoplasmic SPTAN1 was related to the proliferative and invasive capacity of cells and suggested as a marker for neoplasia (Figure 2(b)) [28, 36, 39, 57, 58]. The impact of SPTAN1 on proliferation could be confirmed by* in vitro* data of our group showing a clear decrease of cell proliferation in SPTAN1-deficient cells [30]. The exact molecular mechanism, however, is yet unclear. Loss of membranous SPTAN1 leads to loss of cell polarity, which is the prerequisite for depolarization and proliferation and might therefore induce cell growth [39]. In this case, various SPTAN1-containing membrane complexes including spectrin-actin crosslinks and the SPTAN1-ankyrin-protein 4.1-adducin complexes might be affected, leading to alteration in membrane trafficking, cell signaling, and adhesion complexes and to mechanically fragile cell membranes [17, 18, 59, 60]. Furthermore, cytoplasmic SPTAN1 could interact with different partners and undergo distinct posttranslational modifications. Phosphorylation of SPTAN1 at tyrosine Y1176 might, for example, activate cellular mechanisms that allow or promote SPTAN1 breakdown (Figure 1) [61]. Besides increased cytoplasmic localization, a second pathological change for SPTAN1 has been described: increased abundance of SPTAN1 especially in the apical regions of epithelial cells, which might lead to enhanced extension of core actin bundles into the cytoplasm and a thickened web [28]. Otherwise, SPTAN1 could be located in more central cytoplasmic regions by actin bundles. This may promote cell growth as well but also enables transport of SPTAN1 and other interacting proteins into the cytoplasm, where it might act as a “switchboard” [12].

How the enhanced cytoplasmic SPTAN1 level is managed by the cell is still not clear. In erythroleukemic cells, SPTAN1 has been shown to be induced by treatment with DMSO followed by rearrangements into submembranous patches and caps [49]. DMSO was also described to induce cardiac differentiation in P19 embryonal carcinoma stem cells; however, this mechanism also remains unresolved [62]. Another mechanism influencing the localization of SPTAN1 might be based on the cellular ionic environment. Depending on calcium levels, SPTAN1 has been shown to change its localization in keratinocytes [57]. Under standard levels of calcium, SPTAN1 was concentrated along the cell margin, whereas a low calcium level led to SPTAN1 dissemination throughout the cell and most interestingly a more rapid proliferation of cells [57]. Therefore, SPTAN1 might be able to influence cellular proliferation by calmodulin or calcium binding. This speculation is confirmed by Perrin et al. who demonstrated that calcium-induced cell depolarization seems to be the calmodulin-dependent stimulus initiating patch formation by SPTAN1 in secretory cells [63]. SPTAN1 associated with membrane-bound actin filaments was also shown to be hydrolyzed by a Ca^2+^-dependent protease during platelet activation, a mechanism leading to cell congregation and adhesion [64]. Furthermore, the SPTAN1 interacting protein calmodulin binds calcium and can have regulatory functions of cytoskeletal integrity by activating other proteins including calcium-dependent proteases accelerating degradation of SPTAN1 [65]. Also sodium/potassium homeostasis could have influences on SPTAN1, as Na/K-ATPase and cytoskeletal proteins including SPTAN1 and ankyrin are accumulated at regions of cell-cell contact [66, 67].

Furthermore, enhanced SPTAN1 expression has been shown to be associated with invasiveness and more aggressive tumors [28, 36, 40]. In this context, SPTAN1 seems to also influence cell mobility and invasion. Our group demonstrated SPTAN1-dependent cell migration in CRC cells which confirms this finding [29, 30]. Similar to cell surface proteins of nonneuronal cells, SPTAN1 was described to be involved in crosslinking as well as the cap formation process [68]. As cap formation is a characteristic of moving cells, SPTAN1 could strengthen cell motility via actin-based motility or via an actin-independent mechanism [69]. Upon cell-cell contact, SPTAN1 interacts with ankyrin and the cell adhesion molecule E-cadherin located on membranes and brush borders where it might facilitate cell adhesion [67]. A gradual assembly of ankyrin-spectrin based matrix at sites of E-cadherin-induced cell-cell-contact may also involve recruitment of ankyrin-spectrin-complexes linked to other integral membrane proteins including Na/K-ATPase [67]. These processes are of high interest for epithelial-mesenchymal transition (EMT). In EMT, which is an important feature in cancer initiation and metastasis by enabling migration, calcium-dependent cadherins like E-cadherin are involved. In order to understand if SPTAN1 is essential for cytoskeletal integrity it would be interesting to analyze, on one hand, whether the loss of SPTAN1 affects the cytoskeletal interacting proteins including actin, ankyrin, adducin, protein 4.1, and calmodulin. On the other hand, interacting proteins like E-cadherin involved in EMT processes are of high interest.

During tumor growth and development, cell survival under low oxygen conditions and angiogenesis become more and more important. Interestingly, hypoxia-induced splicing of SPTAN1 has been described in endothelial cells, suggesting a role in angiogenesis-mediated cytoskeletal remodeling [70]. This is probably facilitated by a longer SPTAN1 isoform harboring 20 additional amino acids (aa) C-terminal to the SH3 domain, which is specifically localized at gap junctions and associates with connexin 43 (Cx43) (Figures 1 and 2(c)) [70, 71]. This binding is dependent on the 20 aa insertion which may be sensitive for phosphorylation by the c-Jun N-terminal kinase (JNK) and thus regulated by JNK signaling [70, 71]. Under hypoxia, generation of this spliceform is repressed, resulting in expression of a shorter isoform lacking the specific 20 aa insertion, and therefore reduced gap junction formation. Hence, Weigand et al. [70] suggested that alternative splicing of SPTAN1 contributes to cell survival and angiogenesis.

As already mentioned above, breakdown products of SPTAN1 were found during apoptosis [23, 55]. Redistribution and polar aggregation of SPTAN1 together with PKC*θ* is an early event of apoptosis and has been suggested as a tool to monitor cell death efficiency [72]. In addition, the proteolysis of SPTAN1 during apoptosis has been shown to be dependent on the protooncogene c-myc [73]. SPTAN1 can be cleaved by calpain leading to a 150 kDa fragment [74]. This might be regulated by (de)phosphorylation of the residue Y1176 near the calpain cleavage site by tyrosine kinase src or the low-molecular-weight phosphotyrosine phosphatase A [61, 75]. In prostate cancer cells, SPTAN1 cleaved by calpain induced apoptosis upon treatment with the anticancer drug bicalutamide [76]. However, the cleavage of SPTAN1 by calpain does not necessarily lead to cell death but rather has regulatory functions in secretion and activation under physiological conditions [74]. SPTAN1 breakdown products were also shown to promote adhesion focal disruption, cell rounding, and detachment in epithelial cells [77]. Moreover, dephosphorylation of Y1176 could enhance the proteolytic susceptibility of SPTAN1 to apoptosis associated caspases including caspases 2, 3, and 7 and cleavage leading to cell shrinkage, membrane blebbing, and irreversible cell death (Figure 1) [55, 78, 79]. This is in turn inhibited by calmodulin binding and indicates a high influence of calcium homeostasis [78]. By an independent mechanism through a yet unknown caspase, TGF*β* is also able to induce SPTAN1 cleavage and apoptosis [80].

Altogether, SPTAN1 is involved in apoptosis through cleavage by calpain and caspases. It is conceivable that SPTAN1 bypasses degradation and thus enables tumor cells to evade apoptosis due to overexpression and altered localization of SPTAN1 or altered binding properties to interacting proteins. In ovarian cancer cells, inhibition of SPTAN1 cleavage and apoptosis has been described via an alternatively spliced caspase 2 short isoform (casp-2s) [81]. Casp-2s inhibits DNA-damage induced cytoplasmic SPTAN1 cleavage independent of p53 status and prevents cisplatin-induced membrane-blebbing. Via this pathway, tumors may not only obviate apoptosis but also evade chemotherapy, as described later.

Cell rounding and detachment are important first steps in metastasis. Interestingly, cleavage of SPTAN1 by the enteropathogenic* E.coli *gene EspC leads to sequential degradation of the focal adhesion proteins paxillin (PXN) and focal adhesion kinase (FAK) and consequently to cell rounding and detachment [77]. By mechanical coupling of the actin cytoskeleton to a substrate via focal adhesions, cells are anchored to the extracellular matrix. By disrupting this mechanism, SPTAN1 could have influences on cell detachment, apoptosis, and migration of cancer cells as described for PXN and FAK, both of which are also described to be upregulated in several cancers [82, 83].

Interestingly, SPTAN1 was mapped near the translocation breakpoint region on chromosome 9 in the Abelson murine leukemia (ABL) protooncogene which is involved in the formation of the Philadelphia chromosome in leukemia [84–86]. Since it was mapped centromeric to the breakpoint, it is not translocated to chromosome 22 in the human chronic myelogenous leukemia cell line K562 [85]. However, mutations or fusions including SPTAN1 might also affect ABL and multilayered downstream signaling. Moreover, SPTAN1 is involved in the regulation of cell-cell contact in immunological synapse formation in T cells, pointing also to potential immunological effects [87].

### 3.2. SPTAN1 as a Tumor Suppressor

Until now, downregulation of SPTAN1 was described only in lung cancer, in a lung metastasis of prostate cancer, and in MMR-deficient CRC [29, 34, 38]. Suppression of SPTAN1 expression could be executed by microRNA-128-3p as shown in lung cancer cells [16]. Interestingly, targeting of SPTAN1 led to reduced protein levels, induction of cell cycle arrest, chromosomal instability, and limited DNA repair [16]. Therefore, physiological SPTAN1 recapitulates several tumor-suppressing characteristics, whereas alteration in regulation and localization can lead to differential tumor promoting effects.

A role for SPTAN1 as a tumor suppressor is indicated by the decline of SPTAN1 in tumor-prone FA patients and in MMR-deficient CRCs [29, 88]. FA is a genetic disorder with impaired DNA repair function, bone marrow failure, and an increased risk to develop cancer. Interestingly, SPTAN1 is lacking in cells from FA patients [89–91] and knockdown of SPTAN1 leads to chromosomal instability and impaired interstrand cross-link repair, an effect also observed in FA cells [92]. However, FA cell lines exhibit only reduced SPTAN1 protein levels, whereas mRNA expression remains unchanged, suggesting a regulation at the protein level by stabilization or degradation [93]. In the nucleus (Figure 3), SPTAN1 is stabilized through binding to FA proteins in nuclear complexes and was suggested to act as a scaffold to align or enhance DNA repair associated proteins at sites of damage [25, 89, 90]. One of these FA proteins, FANCG, was described to bind SPTAN1 by its SH3 domain, which seems to be necessary for binding also in FA proteins (Figure 1) [94]. This role for SPTAN1 is further strengthened by the finding of nuclear SPTAN1 enabling DNA repair of interstrand crosslinks (ICL) via FANCA and XPF [95]. Furthermore, SPTAN1 circumvents telomere dysfunction after ICL damage by a related mechanism and interaction with TRF1/2 and XPF [88].

As already mentioned, we have also shown that SPTAN1 is reduced in tumors and cell lines deficient in the MMR protein MLH1 [29, 30]. Accordingly, our group has previously demonstrated a direct interaction of SPTAN1 and MLH1 in the nucleus [47]. In sporadic MLH1-deficient CRC and in MLH1-deficient hereditary Lynch syndrome, SPTAN1 was shown to be reduced as well, which in addition affected cell viability, mobility, and migration* in vitro* [29, 30]. Of note, FANCJ also interacts with the MMR complex MutL*α* consisting of MLH1 and PMS2 by binding directly to MLH1 through a helicase domain, and this interaction is required for correction of the cross-link response [96]. As Peng et al. suggested, this functional connection of FA and MMR predicts a broader role in damage signaling independent of BRCA1 and might represent a so far unknown repair mechanism involving SPTAN1, MLH1, and FA proteins [96]. Until now, SPTAN1-dependent DNA repair signaling pathways are not known in detail. To further elucidate how SPTAN1 is translocated to the nucleus, which isoforms are involved, and which specific functions SPTAN1 has in the nucleus, in DNA repair and potential other mechanisms including chromatin remodeling, a lot of work still has to be done [24].

Furthermore, the tumor suppressing properties of SPTAN1 might be regulated throughout the cell and at plasma membranes. In noninvasive squamous carcinoma cells, SPTAN1 was detected in podosomes, whereas it was absent in invasive invadopodia, suggesting a tumor preventing role [97]. In addition, members of the spectrin-ankyrin-adducin membrane skeleton were implicated as tumor suppressors [17]. Protein 4.1R was described to suppress meningioma pathogenesis and 4.1B has growth suppressing properties in lung cancer and meningioma as well [98–100]. Furthermore, SPTAN1 was shown to interact with Rho GTPase-activating protein and tumor suppressor Deleted in liver cancer 1 [101]. Hence, SPTAN1 itself could not only act as a tumor suppressor in the nucleus but also throughout the cell cytoplasm and furthermore stabilize other tumor suppressors as described above.

Besides the downregulation of SPTAN1 in lung cancer compared to normal tissue, Sun et al. described conserved recurrent gene mutations in both the SPTAN1 gene and mRNA, which were highly correlated with pathway deregulation and worse survival [34]. The reduced SPTAN1 expression may account for its compromised DNA repair capacity and other tumor promoting properties. Sun and coworkers could not find differences in SPTAN1 expression by comparing tumor with and without mutations [34]. However, it is unclear whether these mutations cause a change in the expression of different isoforms or affect protein stability by posttranslational modifications, as this was not analyzed in the study. A gene mutation may not necessarily cause a change in the expression level but can lead to abnormal interactions with other proteins and therefore influence associated pathways or networks [34]. Enriched pathways for SPTAN1 from exome sequencing included Sertoli cell junction signaling (SPTA1 was identified here as well) and apoptosis signaling [34]. In addition, mutated SPTAN1 was mapped in a closely related interaction network of “Cancer, gastrointestinal disease and respiratory disease” including caspase, FAK, JNK, and TP53 and suggesting mutual influence [34].

Recurrent mutations identified in SPTAN1 were single nonsynonymous nucleotide mutations, namely, C→T alterations in exons 30 and 31 and G→C substitutions in exons 37 to 39 [34]. Mutations and deletions in SPTAN1 have been described earlier in disease including intellectual disability, early-onset dystonia, and epileptic encephalopathy as well as multisystemic vascular dysplasia [102–108]. It was suggested that especially in-frame mutations of SPTAN1 may exert a dominant-negative effect by inducing aggregation of defective spectrin subunits and heterotetramers causing instability of various proteins and of the transport machinery [103, 106, 108]. As the last two spectrin repeats are required for heterodimer formation, mutations in this C-terminal region of SPTAN1 are critical and might have major consequences. In addition, Gartner et al. [107] suggested that deleterious variants of SPTAN1 may cause reduced mRNA expression as observed in two unique SPTAN1 variants.

### 3.3. Potential E2/E3 Enzymatic Activity of SPTAN1

Posttranslational ubiquitination depending on the type and number of ubiquitin bonds can regulate the half-life and function of proteins as well as their localization within the cell. Ubiquitin-protein-ligases catalyze the transfer of ubiquitin to a protein. Highly interesting in connection with the regulation of SPTAN1 function is the discovery of an E2/E3 ubiquitin-protein-conjugating/ligating activity that allows self-ubiquitination in erythrocyte spectrins [109–111]. Goodman et al. suggested also an E2/E3 activity for nonerythroid spectrins including SPTAN1 due to sequence homology and conservation of specific cysteines in the spectrin C-terminal repeat 20 (Figure 1) [111]. As SPTAN1 is expressed throughout development, found in various cell compartments, associated with various cytoskeletal components as well as cell adhesion proteins and can target by indirect interaction a multitude of other proteins including transporters and channels, the enzymatic ability of ubiquitination might have a broad impact on the cell and therefore is intriguing to explore [111]. Depending on the type and extent of ubiquitination, SPTAN1 could be precisely regulated and would be self-regulating according to its location, function, and degradation.

## 4. SPTAN1 in Therapy Outcome and Chemoresistance

SPTAN1 seems to play a role in therapy outcome and chemoresistance as well. This is illustrated by a gene expression study of ovarian tumors obtained before and after adjuvant chemotherapy (CT) by L'Esperance et al. [41]. Here, SPTAN1 levels were increased in post-CT ovarian cancer and SPTAN1 was classified as a tumorigenic gene [41].

In an elegant systems medicine approach, carried out by Ajorloo et al., autoantibody profiling was employed to identify target proteins affecting treatment outcome in patients with non-Hodgkin lymphoma [43]. This study identified SPTAN1 amongst others as a hub in patients who underwent chemotherapy and as a key protein for therapy outcome [43]. Indeed, SPTAN1 could be linked to chemoresistance and different reactome pathways including VEGFR2 mediated cell proliferation, Erbb2, and PDGF signaling. Changes in spectrin organization were also described earlier in lymphoid (non-Hodgkin lymphoma) and leukemic (acute lymphoblastic leukemia) cells upon chemotherapy [44]. Here, membrane-associated spectrin remained unchanged, whereas increased SPTAN1 appeared as a dense spectrin network mainly in the area of the nucleus [72]. However, chemotherapeutical response of cisplatin-treated triple-negative breast cancer (TNBC) cells was detected to be associated with SPTAN1 cleavage [112]. The data suggest that cisplatin-dependent activation of calpain 1 in TNBC cells induces an increase of calcium and calmodulin by endoplasmic reticulum stress, whereupon SPTAN1 and caspase 12 are cleaved, which then leads to apoptosis [112]. One therefore might assume that TNBC cells' resistance to cisplatin might be caused by the lack of calpain 1 activation and the lack of spectrin cleavage. Hypothetically, one might also suppose that the induction of SPTAN1 cleavage by drugs might be a promising approach to sensitize cisplatin-resistant TNBC cells. Thus, SPTAN1 or its cleavage products might also be useful as markers of apoptotic tissue and might give hints on therapeutic efficacy.

The anticancer drug bicalutamide, clinically used in prostate cancer patients, has been shown to enhance SPTAN1-mediated apoptosis by calpain or caspase 3 leading to cell shrinkage and membrane blebbing [76]. SPTAN1-mediated apoptosis by anticancer drugs has been also described in lung and hepatocellular carcinoma cells [76, 113]. This therapeutic approach might be of special interest for SPTAN1 overexpressing cancers, as SPTAN1 degradation is involved in the canonical pathway and might foster tumor cell death.

Interestingly, the SPTAN1 interaction partner MLH1 has been also shown to enhance sensitivity to cisplatin by activating apoptosis via a MLH1/ABL signaling pathway [114]. Besides PARP, caspases 3 and 9 are involved in this pathway [114] and suggest a yet unknown role for SPTAN1 in drug sensitivity.

SPTAN1 suppression by microRNA-128-3p led to enhanced sensitivity to cytostatic MMC by limiting DNA repair in lung cancer cells which could be applied for adjuvant chemotherapy in lung cancer [16]. However, microRNA-128-3p might represent a double-edged sword, as it was identified as a novel oncogenic miRNA targeting the tumor suppressor PHF6 gene in T-cell acute lymphoblastic leukemia [115]. Hence, in order to enhance the cytotoxic effects of MMC treatment, it might be reasonable to target SPTAN1 in cancer directly. However, this requires further clarification and analysis of the mode of action and involved partners.

Currently, new approaches to target CRC stem cells are under investigation to improve treatment of severe CRCs, as recently reviewed by Thenappan et al. [116]. Interestingly, Wnt and transforming growth factor-*β* (TGF-*β*) signaling pathways were described to regulate stem cell function and influence cancer [117], both of which can be potentially influenced by spectrins. SPTBN1, also termed embryonic liver fodrin (ELF), can activate and modulate TGF-*β* by Smad activation as found in an SPTBN1-deficient mouse model [118]. Furthermore, SPTBN1 expression is reduced in early stage CRC and that of Smad4 in advanced carcinomas, which indicates a key role for SPTBN1/Smad4/TGF-*β* signaling in the suppression of cancer progression [119]. Therefore, association of SPTBN1 with SPTAN1 is conceivable, as different SPTBN1 and SPTAN1 levels could mutually influence each other. Finally, SPTAN1 could also influence Wnt signaling by interacting with E-cadherin in an E-cadherin/ankyrin/SPTAN1 complex and thus modulate *β*-Catenin/Wnt but also other signaling pathways [67].

## 5. Conclusions

SPTAN1 plays an important role in cancer development and progression. The expression level of SPTAN1 is enhanced in several tumors, while it is decreased in others, and its expression has been associated with progression of disease and metastasis. SPTAN1 might serve as both a tumor suppressor and promotor by several mechanisms. Its tumor suppressing characteristics include maintaining cell shape and cytoskeletal architecture by interacting with other cytoskeletal proteins and enabling DNA repair. SPTAN1's switchboard and transporter function as well as its role in apoptosis, EMT, adhesion, and migration can influence tumor growth and progression in both positive and negative directions depending on the specific regulation. Specifically, enhanced cytoplasmic SPTAN1 is associated with neoplasia and progression. Therefore, SPTAN1 can have a broad impact on cell dynamics and tumor development. Most importantly and promising for clinical practice, SPTAN1 has the potential to be used as a tumor marker for progression as well as a marker for therapy decisions. In this regard, the following features are particularly important when considering its relevance: (a) cellular localization, (b) level of expression in tumors, (c) isoforms, and (d) posttranslational modifications.

SPTAN1 is comprehensively involved in cell dynamics and a better understanding of its mechanism of action and fine tuning in various processes leads not only to a better understanding of tumor characteristics but also beyond in general cell organization and regulation processes.

## Figures and Tables

**Table 1:** SPTAN1 in various cancer types.

Tumor Type	Changes in SPTAN1	References
Colorectal cancer (CRC)	SPTAN1 upregulation	[28]
	SPTAN1 downregulation in MLH1-deficient CRC	[29]
	SPTAN1 upregulation in sporadic CRC, SPTAN1 downregulation in MLH1-deficient CRC	[30]

Gastric cancer	SPTAN1 upregulation	[28, 31, 32]

Lung cancer	SPTAN1 upregulation	[33]
	SPTAN1 mutations and downregulation	[34]

Breast cancer	SPTAN1 upregulation (cytoplasmic)	[28, 35, 36]

Bladder cancer	Recurrence-associated SPTAN1 alterations	[37]

Prostate cancer	SPTAN1 downregulation in lung metastasis, SPTAN1 as progression gene	[38]

Cutaneous tumors	SPTAN1 upregulation (cytoplasmic)	[39]

Soft-tissue tumors	SPTAN1 upregulation in more aggressive mesenchymal tumors	[40]

Ovarian cancer	SPTAN1 upregulation after chemotherapy	[41]

Atypical chronic myeloid leukaemia (aCML)	Novel CSF3R-SPTAN1 fusion gene	[42]

Non-Hodgkin lymphoma	SPTAN1 as target protein in postchemotherapy	[43]

Non-Hodgkin lymphoma, acute lymphoblastic leukaemia (ALL)	SPTAN1 upregulation after chemotherapy in nuclear area	[44]

## Abbreviations

Aa: Amino acid
ABL: Abelson murine leukemia
aCML: Atypical chronic myeloid leukemia
CaM: Calmodulin
Casp-2s: Caspase 2 short isoform
CDP-1: Calcium-activated protease 1, calpain
CRC: Colorectal cancer
CSF3R: Colony-stimulating factor 3 receptor
CT: Chemotherapy
Cx43: Connexin 43
DMSO: Dimethyl sulfoxide
ELF: Embryonic liver fodrin
EMT: Epithelial-mesenchymal transition
EVL: Ena/vasodilator-stimulated phosphoprotein-like protein
FA: Fanconi anemia
FAK: Focal adhesion kinase
JNK: c-Jun N-terminal kinase
MMC: Mitomycin C
MMR: DNA mismatch repair
PXN: Paxillin
SFT: Soft tissue tumor
SH3: Src homology domain 3
SPTA1: Erythroid spectrin *α*I
SPTAN1: Nonerythroid spectrin *α*II
SPTBN1: Nonerythroid spectrin *β*II
TNBC: Triple-negative breast cancer
TGF: Transforming growth factor
VASP: Vasodilator-stimulated phosphoprotein
Y: Tyrosine.
